# Polydopamine-Assisted Two-Dimensional Molybdenum Disulfide (MoS_2_)-Modified PES Tight Ultrafiltration Mixed-Matrix Membranes: Enhanced Dye Separation Performance

**DOI:** 10.3390/membranes11020096

**Published:** 2021-01-30

**Authors:** Huali Tian, Xing Wu, Kaisong Zhang

**Affiliations:** 1 Key Laboratory of Urban Pollutant Conversion, Institute of Urban Environment, Chinese Academy of Sciences, Xiamen 361021, China; hltian@iue.ac.cn; 2 University of Chinese Academy of Sciences, Beijing 100049, China; 3 CSIRO Manufacturing, Clayton South, VIC 3169, Australia; Xing.Wu@csiro.au

**Keywords:** tight ultrafiltration membrane, molybdenum disulfide, dopamine, two-dimensional nanomaterial, polyethersulfone

## Abstract

Tight ultrafiltration (TUF) membranes with high performance have attracted more and more attention in the separation of organic molecules. To improve membrane performance, some methods such as interface polymerization have been applied. However, these approaches have complex operation procedures. In this study, a polydopamine (PDA) modified MoS_2_ (MoS_2_@PDA) blending polyethersulfone (PES) membrane with smaller pore size and excellent selectivity was fabricated by a simple phase inversion method. The molecular weight cut-off (MWCO) of as-prepared MoS_2_@PDA mixed matrix membranes (MMMs) changes, and the effective separation of dye molecules in MoS_2_@PDA MMMs with different concentrations were obtained. The addition amount of MoS_2_@PDA increased from 0 to 4.5 wt %, resulting in a series of membranes with the MWCO values of 7402.29, 7007.89, 5803.58, 5589.50, 6632.77, and 6664.55 Da. The MWCO of the membrane M3 (3.0 wt %) was the lowest, the pore size was defined as 2.62 nm, and the pure water flux was 42.0 L m^−2^ h^−1^ bar^−1^. The rejection of Chromotrope 2B (C2B), Reactive Blue 4 (RB4), and Janus Green B (JGB) in aqueous solution with different concentrations of dyes was better than that of unmodified membrane. The separation effect of M3 and M0 on JGB at different pH values was also investigated. The rejection rate of M3 to JGB was higher than M0 at different pH ranges from 3 to 11. The rejection of M3 was 98.17–99.88%. When pH was 11, the rejection of membranes decreased with the extension of separation time. Specifically, at 180 min, the rejection of M0 and M3 dropped to 77.59% and 88.61%, respectively. In addition, the membrane had a very low retention of salt ions, Nacl 1.58%, Na_2_SO_4_ 10.52%, MgSO_4_ 4.64%, and MgCl_2_ 1.55%, reflecting the potential for separating salts and dyes of MoS_2_@PDA/PES MMMs.

## 1. Introduction

Tight ultrafiltration (TUF) membranes have received increasing attention from water treatment and resource recovery in recent years [1]. Especially in the treatment of dye wastewater, the TUF membrane of mesoporous shows more beneficial effects than nanofiltration (NF) membranes, not only because of the relative lower operating pressure required by the TUF process, but also because of the pore size characteristics of TUF membranes. The sieving effect plays a major role. The dye molecules could be intercepted and the salt ions pass through the membrane pores. Higher purity dye would be obtained. However, due to the combined effect of the sieving effect, dissolution diffusion, and Donan effect [2], traditional nanofiltration (NF) membranes often have a high retention rate of divalent salt ions, which will increase the difficulty of further separation of salt and dye. The TUF membrane is actually a type of ultrafiltration membrane (UF) with a molecular weight cut-off (MWCO) of 1000–10,000 Da and pore size of ≈2–10 nm. To achieve the small pore size, a further interfacial polymerization process [3], surface coating [4], surface deposition [5] and membrane porous filling [6] are applied on as-prepared UF membranes. However, considering the limitations of mass production, phase inversion is still the first choice. Without complex polymer modification and complicated preparation procedures, blending with nanoparticles and polymer to prepare a mixed matrix membrane (MMM) is one of the important means to improve the performance of the membrane. However, poor compatibility between nanoparticles and polymers may provide the non-selective voids and have negative impacts on membrane properties and performance such as reducing the retention rate.

To enhance the compatibility between nanoparticles and polymers, dopamine (DA, 3,4-dihydroxyphenethylamine) has become a popular choice in modification research. Inspired by the mussel’s adhesion behavior, DA has been extracted and applied successfully as glue to the surface modification of various organic and inorganic substances [7]. The application of DA has become a promising membrane modification strategy due to its wideness and versatility [8]. DA self-polymerization forms thin and surface-adherent polydopamine (PDA) films on the membrane surface through deposition. It has been reported that the PDA coating could improve the surface hydrophilicity of PES, polyvinylidenefluoride (PVDF), polysulfone (PSF), polytetrafluroethylene (PTFE), and polyethylene (PE) membranes, and enhance the antifouling ability of these membranes [9,10,11]. In addition to the coating, another method of adding DA to the membrane is blending. The blending method can be performed by introducing DA or PDA to the membrane casting solution directly [12]. This method improves the hydrophilicity, permeability, and antifouling performance of the membrane, increases the porosity and pore size of the membrane. However, the increase in porosity brings great defects to the membrane, such as permeate purity and mechanical properties [13]. In some cases, PDA-encapsulating nanoparticles were blended into the polymer mixed matrix to improve the membrane performance. PDA builds up an intermediate layer around the surface of nanoparticles, enhances the interface compatibility, and reduces the defects of MMMs [14]. Ndlwana et al. blended PDA-crosslinked graphene nanoplatelets (GNP-PDA) to polyethersulfone (PES) MMMs for improved mechanochemical properties [15]. Zhang et al. blended PDA-coated metal–organic frameworks (MOFs) into hydrophilic polymer polyvinyl alcohol (PVA) to enhance the water permeability and selectivity of the MMMs [16]. Sianipar et al. found that the incorporation of PDA-coated multi-walled carbon nanotubes (MWNTs) could increase the membrane permeability without a reduction of selectivity as well as improve the antifouling properties, and enhanced mechanical strength was also found by adding PDA-coated MWNTs into membranes [17]. Wu et al. blended TiO_2_@PDA into the PSF casting solution to prepare a UF membrane with catalytic properties. PDA as a free radical scavenging agent improved the antifouling ability of the membranes [18]. Most of these membranes focus on anti-pollution performance, and PDA@NPS has seldom studied the MWCO change in membranes. Molybdenum disulfide (MoS_2_) is a graphene-like material with a relatively large area. It performs well in RO, NF, and UF membrane and improves the permeation without sacrificing retention [19,20,21].

In this work, polydopamine-modified two-dimensional MoS_2_ was used as a novelty additive to regulate the MWCO of the TUF membrane. The impact of MoS_2_@PDA on PES-based TUF membranes was investigated by fabricating a series of MoS_2_@PDA/PES mixed matrix TUF membranes via the simple phase inversion method. In the optimized MoS_2_@PDA concentration conditions, the MoS_2_@PDA/PES membrane has better stability, smaller pore size, and better dye selectivity than pristine membrane. The morphology structure, surface characteristics, membrane thermostability, dyes separation, and salts filtration performance of the prepared membranes were investigated.

## 2. Experimental

### 2.1. Materials

Commercial polyethersulfone (PES) Ultrason E6020P was supplied by BASF (BASF, Corp., Ludwigshafen, Germany). Molybdenum sulfide (MoS_2_) (99.5% metals basis, <2 μm) (Aladdin, Inc., Shanghai, China). N,N-Dimethylacetamide (DMAc) and Polyvinylpyrrolidone (PVP K30) (Sinopharm, Inc., Shanghai, China). DA hydrochloride, Polyethylene glycol PEG (2000, 4000, 6000, 8000, 10,000 Da), Chromotrope 2B (C2B) (513.37 Da), Reactive Blue 4 (RB4) (637.43 Da), and Janus Green B (JGB) (511.06 Da) were acquired from Sigma Aldrich (Sigma–Aldrich, Inc., St. Louis, MO, USA) Deionized (DI) water was used in all experiments.

### 2.2. Synthesis of MoS_2_@PDA

**Figure 1:**
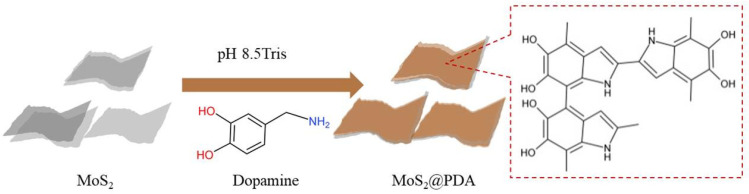
Synthetic scheme of MoS_2_@PDA (polydopamine) nanoparticles.

To prepare MoS_2_@PDA (Figure 1), 0.5 g of MoS_2_ sheets were dispersed in 100 mL of 10 mM Tris-buffer (pH ≈ 8.5) by ultrasonic for 4 h to form a suspension solution. Then, 0.3 g DA hydrochloride was added to the suspension solution, stirred rapidly for 30 min, and then changed to low-speed agitation for another 24 h at normal temperature. Subsequently, the mixing solution was centrifuged, and the product was washed three times with DI water and ethanol, and then dried in an oven at 60 °C. This product was labeled as MoS_2_@PDA.

### 2.3. Membrane Preparation

Table 1 lists the components of different casting solutions. Different contents of MoS_2_@PDA powders were added into the DMAc solution and sonicated for 30 min to form the MoS_2_@PDA uniform dispersion solution. After that, PVP and PES were added into the MoS_2_@PDA solution, and the mixture was stirred at 60 °C until a homogeneous solution was formed. The casting solution was poured on a non-woven fabric on a glass plate. A casting knife gap setting of 200 μm was applied to control the thickness of membranes, and the casting process was conducted under a temperature of 25 °C and humidity of 45 ± 5%. After casting, the plate was immersed into a DI water bath. Finally, the prepared membrane was transferred to fresh pure water for 24 h to remove the residue and then stored in DI water prior to use.

### 2.4. Characterization of MoS_2_@PDA

The morphological structure of MoS_2_ and MoS_2_@PDA was characterized by field emission transmission electron microscopy (FETEM, Tecnai F20-, FEI Corp., Portland, OR, USA). The hydrophilicity of nanomaterials was determined by a contact goniometer by dropping 2.0 µL of water (CA, DSA100, Krüss Company, Ltd., Hamburg, Germany). The zeta potential of MoS_2_ and MoS_2_@PDA was measured using zeta potential (Zeta PALS, Malvern Instruments Ltd., Malvern, UK). The pH was adjusted in the range of 3 to 11 by NaOH and HCl solution. Thermogravimetric analyses (TGA, Perkin Elmer) were conducted under nitrogen atmosphere with a flow rate of 60 mL min^−1^. The sample was placed in a ceramic crucible; the heating temperature ranged from 40 to 1000 °C at a heating rate of 10 °C min^−1^. The crystal structure was tested by X-ray diffraction (XRD, X’Pert Pro, PANalytical, Netherlan) at a scanning rate of 10° min^−1^ in the 2θ range of 10–70°. The chemical structure of MoS_2_ and MoS_2_@PDA was analyzed by Fourier transform infrared spectroscopy (FTIR, Nicolet iS10, Thermo Fisher Scientific Inc., Waltham, MA, USA). X-ray photoelectron spectrometry (XPS, ESCALAB 250XI, Thermo Fisher Scientific Inc., Waltham, MA, USA) was used to characterize the surface elemental composition of MoS_2_ and MoS_2_@PDA.

### 2.5. Membrane Characterization

To verify the existence of MoS_2_@PDA in the MoS_2_@PDA/PES membranes, field emission scanning electron microscopy (FESEM, HITACHIS-4800, Hitachi Co. Ltd., Tokyo, Japan) was used to observe the structural changes of membrane surface and cross-section at 5.0 kV. Before FESEM observation, the dry membrane sample was sputtered with gold. The surface roughness of membranes was determined with atomic force microscope (AFM, Dimension 3100, Bruker Corp., Santa Barbara, CA, USA). The test area is 5 µm × 5 µm. Average values of roughness for the membrane samples were obtained by measuring three different locations. The surface electrical properties were tested using an electro kinetic analyzer (SurPASS3, Anton Paar, Graz, Austria). The pH was adjusted in the range of 3 to 11 by adding NaOH and HCl solution, and 1 mM KCl solution was used as the electrolyte solution. TGA and XPS were applied for analyzing the characteristics of the as-prepared membranes.

### 2.6. Molecular Weight Cut-Off and Filtration Performance of Membranes

The MWCO of membranes were defined using the different molecular weights of polyethylene glycols (PEGs), which were retained with 90% [22]. The test was performed using a dead-end filtration system (Model 8050, Millipore Corp., Burlington, MA, USA) at 0.1 MPa. First, 50 mL of 1.0 gL^−1^ PEG solution was used as the feed liquid. The filtration process was completed when ≈20% of feed solution (i.e., 10 mL) was filtrated through the membrane. Both the feed and permeate solution were diluted by 10 times, and then, the concentration of each solution was tested by a total organic carbon analyzer (TOC, TOC-LCSH, Shimadzu, Japan) [23]. The PEG rejection was calculated using Equation (1):

(1) $$R\left(\%\right)=\left(1-\frac{C_{p}}{C_{f}}\right)\times 100$$

where *C_p_* and *C_f_* are the PEG concentrations of permeate and feed solutions respectively (gL^−1^). It was reported that the mean effective pore size of the membrane equals the Stokes radius (ds) of PEG at 50% rejection, which could be calculated by Equations (2) and (3) [24]:

(2) $$r_{s}=16.73\times 10^{-12}\times M_{PEG}^{0.557}$$

(3) $$d_{s}=2\times r_{s}.$$

The pore size distribution of the membrane was analyzed by Equation (4).

(4) $$\frac{dR\left(d_{p}\right)}{dd_{p}}=\frac{1}{d_{p}ln\sigma p\sqrt{2\pi }}exp\left[\frac{{\left(lnd_{p}-lnu_{p}\right)}^{2}}{2{\left(ln\sigma p\right)}^{2}}\right]$$

where *r_s_* and *d_s_* stand for the Stokes radius and diameter of the PEG, respectively. *M_PEG_* is the MWCO of PEG. The geometric mean diameter (μs) can be calculated as *ds* corresponding to R = 50%, and the geometric standard deviation (σg) can be determined from the ratio of ds at R = 84.13% and R = 50%. By ignoring the dependence of solute separation on the spatial and hydrodynamic interaction between the solute and the pore size, the average effective pore size (*μp*) and geometric standard deviation (*σp*) of the membrane can be regarded as the same as the value of μs and σg [25,26].

The water flux was measured by a dead-end filtration cell with a volume capacity of 50 mL. The effective area of the membrane was 13.4 cm^2^. Membrane samples were firstly pre-pressed at 0.15 MPa for 30 min, ensuring that the pure water flux reached a steady state; then, we recorded the permeate weight by an electronic balance with Wedge software at 0.1 MPa. The permeate flux (*J*) was calculated with Equation (5):

(5) $$J_{0}=\frac{\Delta V}{A_{m}\Delta t}$$

where $J_{0}$(L m^−2^ h^−1^) is the membrane flux, ΔV(L) is the volume of permeated water, A (m^2^) is the membrane area, and Δt (h) is the permeation time.

The separation performances of the membranes were investigated to filtered dye molecules and salts. The concentration of C2B, RB4, and JGB were measured by Ultraviolet-visible (UV-vis) spectrophotometer (Spectra Max M2, Molecular Devices Co., San Jose, CA, USA). The concentrations of NaCl, Na_2_SO_4_, MgSO_4_, and MgCl_2_ were measured by a conductivity meter (sensION+EC5, HACH). The permeation flux (*J_d_*) and rejection (*R_d_*) of dye were calculated by Equations (1) and (5), respectively.

## 3. Results and Discussion

### 3.1. Characterization of MoS_2_ and MoS_2_@PDA

**Figure 2:**
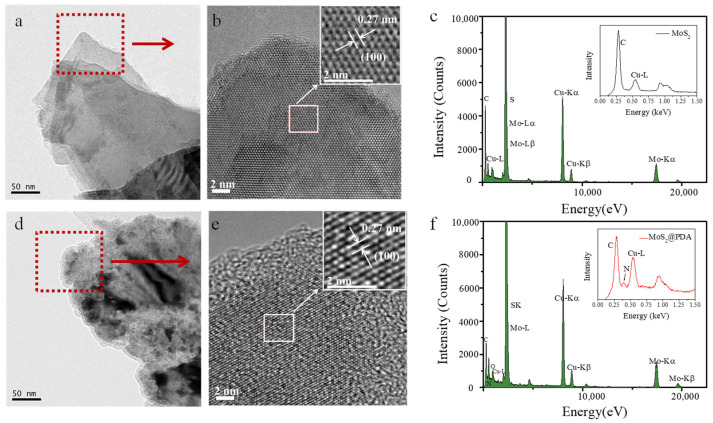
TEM images of MoS_2_ (**a**,**b**) and MoS_2_@PDA (**d**,**e**), EDS spectrum of MoS_2_ (**c**), and MoS_2_@PDA (**f**).

The morphology of MoS_2_ and MoS_2_@PDA was investigated by FETEM (Figure 2). It can be found that the surface of the MoS_2_ was smooth. After modification, MoS_2_@PDA showed many convex coatings in Figure 2d,e. Figure 2b,e show the HRTEM images of MoS_2_ and MoS_2_@PDA. It can be observed that the edge structure of MoS_2_@PDA becomes softened compared to the unmodified MoS_2_. In addition, the inset images revealed that both MoS_2_@PDA and MoS_2_ consist of the hexagonal lattices and the lattice spacing of 0.27 nm, which was corresponding to the (100) lattice plane MoS_2_ [27]. The energy dispersive spectroscopy (EDS) spectrum shown in Figure 2c,f indicated that a new peak N can be detected on MoS_2_@PDA.

**Figure 3:**
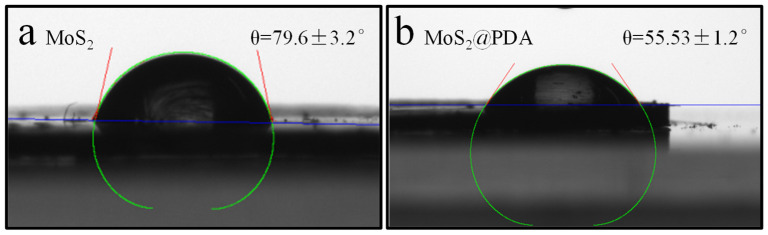
Water contact angles of MoS_2_ (**a**) and MoS_2_@PDA (**b**).

The surface hydrophilicity was characterized by the water contact angle (CA) analysis. Average values were obtained by measuring three different locations. As shown in Figure 3, the CA of pristine MoS_2_ was 79.6 ± 3.2°. Impressively, the CA of MoS_2_@PDA was 55.53 ± 1.2°, which indicated the improved water wettability of MoS_2_ by PDA coating. The major reason for this improvement was that dopamine contains a large number of hydrophilic amines and hydroxyl groups, which polymerize under alkaline conditions to form PDA and deposit around MoS_2_, enhancing the surface hydrophilicity and thus reducing the CA [28,29].

**Figure 4:**
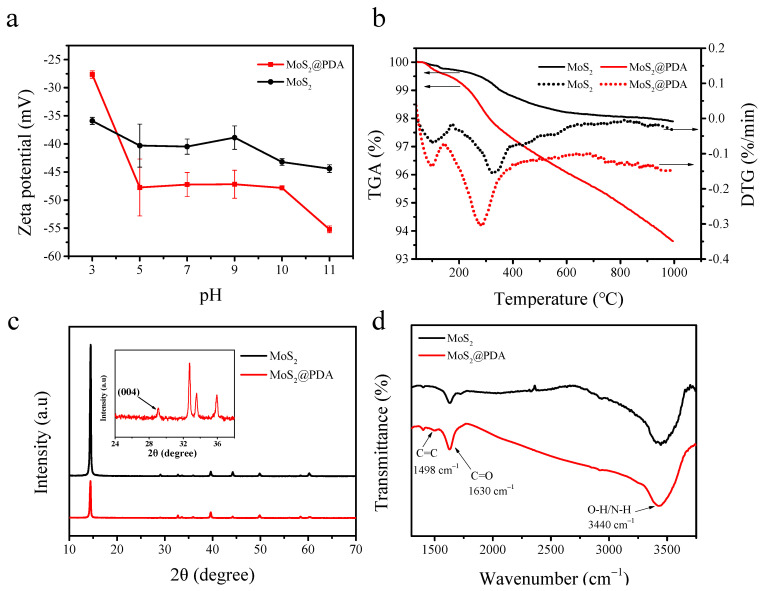
Zeta potential (**a**), Thermogravimetric analyses (TGA)-DTG curves (**b**), X-ray diffraction (XRD) characteristic (**c**), and Fourier transform infrared spectroscopy (FTIR) spectra of MoS_2_ and MoS_2_@PDA (**d**).

The zeta potential characteristic of particles was shown in Figure 4a. Due to the deposition of zwitterionic PDA, at low pH, the amino group of the PDA was protonated. When pH = 3, the potential of MoS_2_@PDA was −27.65 mV, and that of MoS_2_ was −35.9 mV. However, with the increase of pH, the potential of MoS_2_@PDA was lower than MoS_2_. This was due to the negative charge of the PDA phenol group after deprotonation at high pH, which enhanced the negative charge of modified particles [30].

Figure 4b gives the results from TGA and DTG analysis carried out on MoS_2_ and MoS_2_@PDA. There are two distinct stages of mass loss in the TGA curve: ≈100 °C and 100–500 °C, which corresponded to the peaks on the DTG curve at 102 °C, 328 °C for MoS_2_ and 96 °C, 282 °C for MoS_2_@PDA. In the temperature range of 500–1000 °C, the mass loss of MoS_2_ changes slowly, and the mass loss of MoS_2_@PDA was almost a straight line with a slope [31]. The TGA curve of MoS_2_ showed a weight loss of 2.10% when the temperature increased from 0 to 1000 °C. It was mainly due to the weight loss caused by the water or other impurities absorbed on the MoS_2_ [32]. The weight loss rate of MoS_2_@PDA was 6.36%, which was higher than the pristine MoS_2_, indicating that the PDA was coated on the MoS_2_ surface. According to the calculation of the TGA curve of unmodified and modified, the PDA deposition on MoS_2_ sheets was approximately 4.26%.

The crystallographic structures of the pristine MoS_2_ and MoS_2_@PDA composite were characterized by XRD patterns, as shown in Figure 4c. Peaks at 2*θ* = 14.3°, 29°, 39.6°, 44°, 49.8°, and 60.1° were well associated with MoS_2_ (JCPDS card no. 37–1492), which can be assigned to (002), (004), (103), (104), (105), and (110) planes, respectively [33,34]. After encapsulation with PDA, there were no new crystallization peaks. The XRD pattern of MoS_2_@ PDA still had characteristic diffraction peaks at (002), (004), (103), (104), (105), and (110), which showed that before and after PDA wrapped, the crystal structure of MoS_2_ had not changed [35]. However, the existence of PDA affected the crystallinity of the MoS_2_ crystal [27]. The introduction of carbon would lead to the reduction of the diffraction peak [36]. Due to the deposition of dopamine, the carbon element increased, the purity of MoS_2_ crystal decreased, and the intensity of the diffraction peak reduced.

The FTIR spectra of MoS_2_ and MoS_2_@PDA are shown in Figure 4d. Compared to the spectra of MoS_2_, there was a new peak in the spectra of MoS_2_@PDA. The peak at around 1498 cm^−1^ was the C=C skeleton vibration peak of the benzene ring from PDA [37]. The peak at 1630 cm^−1^ was formed by the oxidation of the dobutamine hydroxyl group to the ketone group C=O [38]. The peak at around 3440 cm^−1^ can be attributed to the stretching vibration of N–H and OH [32]. Compared to pristine MoS_2_, the hydrophilicity of MoS_2_@ PDA was enhanced due to PDA deposition, resulting in an enhanced O–H vibration of H_2_O [31].

**Figure 5:**
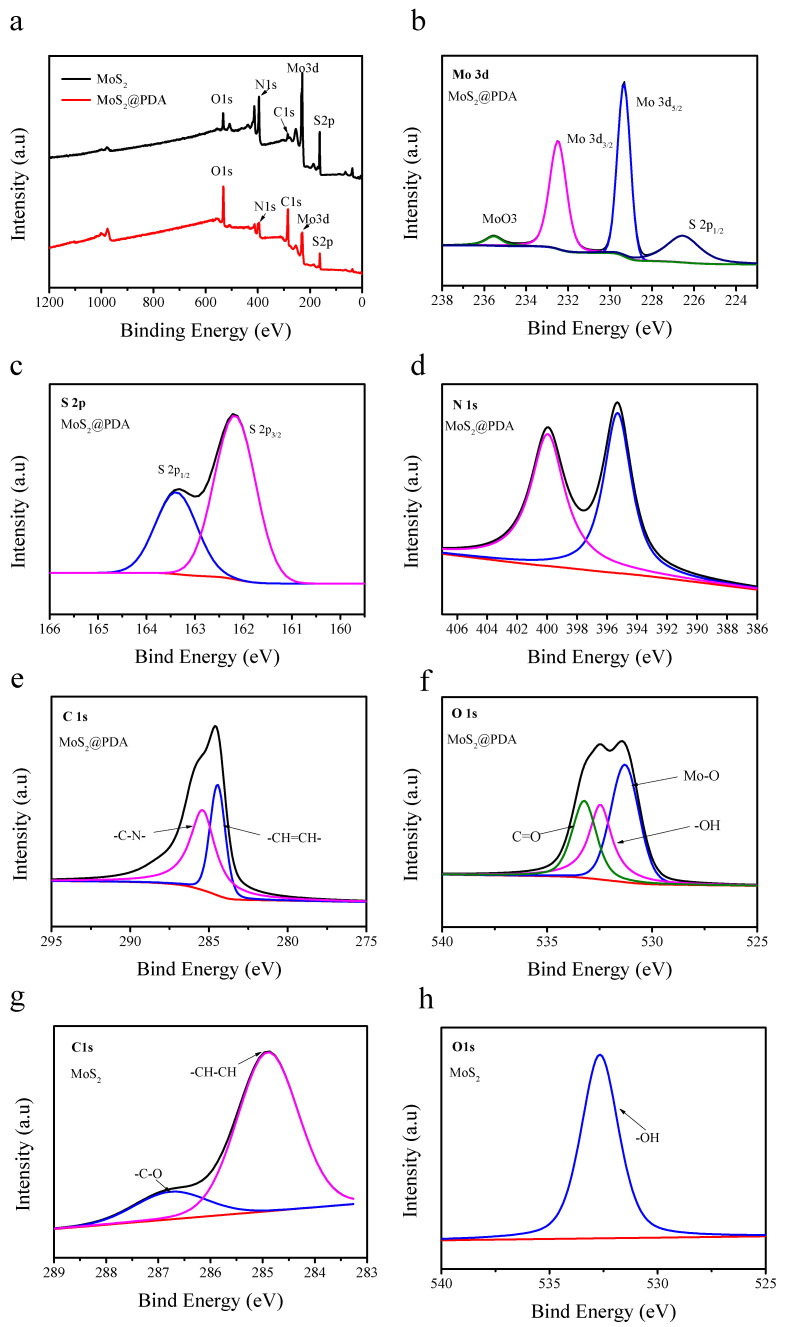
XPS spectra for MoS_2_@PDA and MoS_2_ (**a**), high-resolution XPS spectra of Mo 3d (**b**), S 2p (**c**), N 1s (**d**), C1s (**e**), and O 1s (**f**) for MoS_2_@PDA and C1s (**g**) O 1s (**h**) for MoS_2_.

The chemical composition of MoS_2_@PDA was further investigated by XPS analysis. As shown in Figure 5a, the XPS full energy spectrum investigation indicated that Mo, S, C, N, and O elements were present in the hybrid. It was found that the MoS_2_ characteristic peaks, such as Mo 3d and S 2p, were reduced after the modification of dopamine. However, the intensity of O1s and C1s were enhanced, and we confirmed the production of dopamine-induced polymerization, which was coated on the MoS_2_ surface. The high-resolution Mo 3d spectra (Figure 5b) had three peaks; 229.34 eV and 232.51 eV related to Mo^4+^3d_5/2_ and Mo^4+^3d_3/2_, while the peak at 235.59 eV may be derived from the surface oxidation of Mo^6+^ [36,39]. In the spectra of the high-resolution S 2p (Figure 5c), there were peaks at 162 eV and 163.3 eV corresponding to the bivalent S 2p_3/2_ and S 2p_1/2_ (S^2−^) [40]. There were two peaks in N1 (Figure 5d); the one at 399.9 eV was associated with pyrrolic nitrogen in an indole ring, and the one at 395.3 eV related to the pyridinic resulting from the dopamine functionalization [41,42]. In the MoS_2_@PDA (Figure 5e), the binding energy of C1s at 284.5 eV was assigned to C–C/C–H bonds, while that at 285.7 eV was assigned to C–O/C–N, respectively [43]. In the original MoS_2_ (Figure 5g), C1s at 284.9 eV and 286.8 eV was related to C-C and C–O, which may derived from precursors left over from the formation of MoS_2_. In the analysis of the O 1s, by comparing Figure 5f,h, there were two new peaks in the modified MoS_2_ 531.3 eV, corresponding to Mo–O and 533.23 eV related to the chemisorbed oxygen [44,45]. The binding energy 532.44 eV on MoS_2_ was related to O-bonding with residual water [46].

### 3.2. Characterization of MoS_2_@PDA/PES TUF Membrane

**Figure 6:**
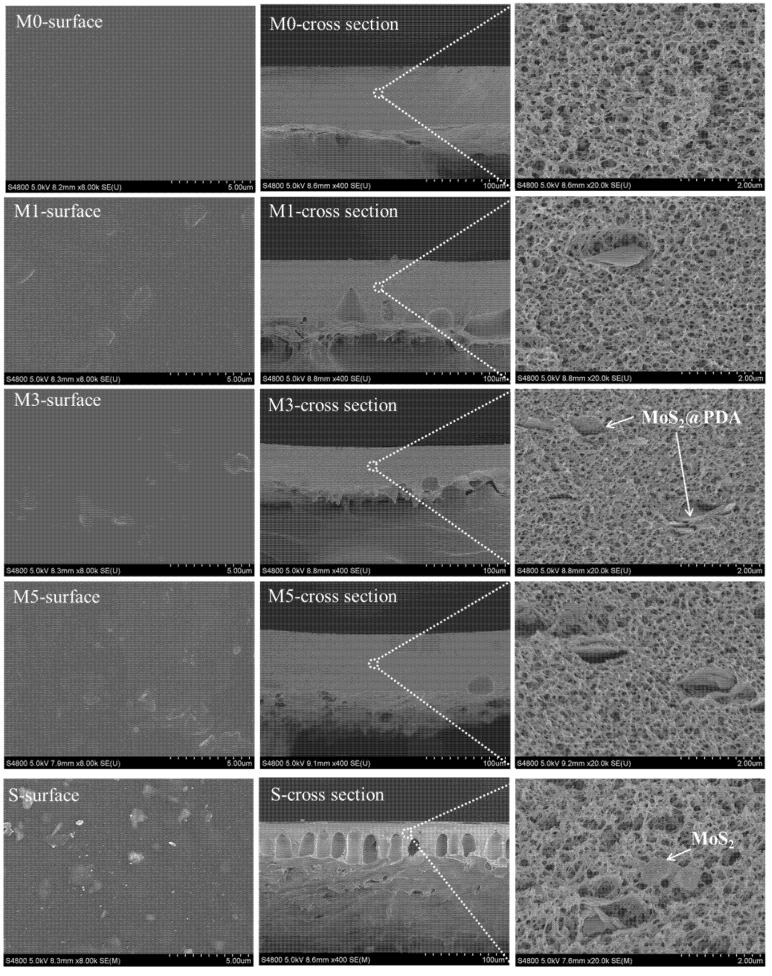
SEM images of PES and MoS_2_@PDA/PES membranes (M0, M1, M3, and M5) and MoS_2_/PES (3.0 wt % of MoS_2_) mixed matrix membrane (S).

The surface and the cross-sectional morphologies of membranes are shown in Figure 6. With the increase of MoS_2_@PDA concentration, the influence of MoS_2_@PDA can be noticed. By adding MoS_2_@PDA, the surface was no longer as smooth as the pristine one. It was because during phase inversion, MoS_2_@PDA migrated from the PES matrix to a water bath and appeared on the membrane surface.

The basic membrane in this study was filled with sponge structures, which was achieved by a high additive amount of PVP [47,48]. This single-layered morphology is favorable for the investigation of the influence of particles in the membrane structure. When the concentration of MoS_2_@PDA was 2.0 wt %, the presence of hydrophilic MoS_2_@PDA in the casting solution accelerated the solvent–nonsolvent exchange and instantaneous liquid–liquid separation in the casting solution, resulting in the formation of the microvoids structures observed in the M1 membrane [49]. However, the number of macropores was reduced at a higher additive concentration, and the internal structure was more compact, as shown in the cross-section of membranes M3 and M5. This was due to the fact that rheology played a leading role in the increase of the viscosity of the casting solution with the addition of MoS_2_@PDA, which reduced the phase conversion rate and densified the membrane structure [50]. The morphological characteristic of the cross-section was different from that of the MoS_2_/PES TUF membrane; the MoS_2_@PDA/PES TUF membrane displays fewer microvoids, and the supporting layer is dominated by a sponge structure. This provides the possibility of forming smaller MWCO membranes.

**Figure 7:**
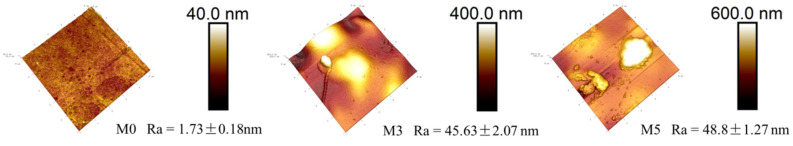
Atomic force microscope (AFM) images of the M0, M3, and M5 membranes.

The surface roughness of membranes was measured by AFM (Figure 7). Results were obtained by measuring three different points and indicated that the M0 membrane displayed a smooth surface with an average roughness (Ra) value of 1.73 ± 0.18 nm. The addition of MoS_2_@PDA made the membrane roughness rise rapidly. When the additive amount was 3.0 wt %, the roughness of the M3 membrane was 45.63 ± 2.07 nm. With the concentration of MoS_2_@PDA increased to 4.5 wt %, the membrane roughness became 48.8 ± 1.27 nm. The membrane surface became rougher. This was related to the hydrophilic MoS_2_@PDA migration to the direction of coagulation bath in the phase inversion process. The increased of membrane surface roughness was consistent with the observation result of SEM.

**Figure 8:**
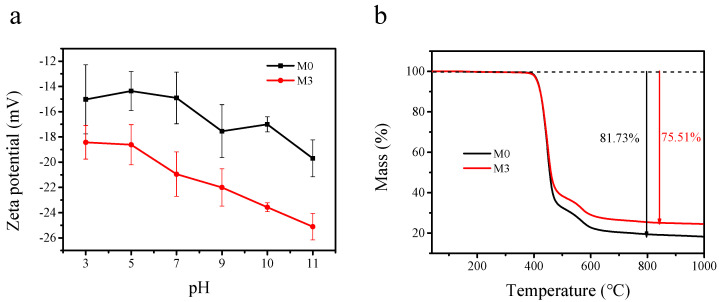
Zeta potential (**a**) and TGA curves (**b**) of pristine membrane and PES/MoS_2_@PDA Membrane.

The surface charge of membranes is a critical factor to affect the filtration performance of membranes. As shown in Figure 8, the pristine M0 membrane showed negative charges ranging from 3 to 11 as the pH values increased. These negative charges were believed to come from the functional groups of PES (O=S=O) and PVP (O=C–N) [51]. After the introduction of MoS_2_@PDA, the M3 membrane showed more negative charges than that of the pristine membrane at different pH values, which was caused by the electronegativity of MoS_2_@PDA.

The TGA analysis was conducted to investigate the thermal stability of the pristine membrane (M0) and the PES/MoS_2_@PDA membrane (M3). As can be seen from the TGA curves in Figure 8, rapid material losses were observed in both the M0 and M3 membrane as the temperature increased from 400 to 600 °C, which was due to the polymer decomposition of PES [52]. When the analysis temperature reached 1000 °C, the weight loss of the M0 membrane was 81.73%, which was larger than the weight loss of the M3 membrane (75.51%). This indicated that the thermal stability of the membrane was improved after the addition of MoS_2_@PDA. This was due to the interaction between the MoS_2_@PDA nanomaterial and PES, which increased the rigidity of the polymer chain and the fracture energy of the polymer chain.

### 3.3. Performance of MoS_2_@PDA/PES TUF Membrane

#### MWCO and Filtration Performance

**Figure 9:**
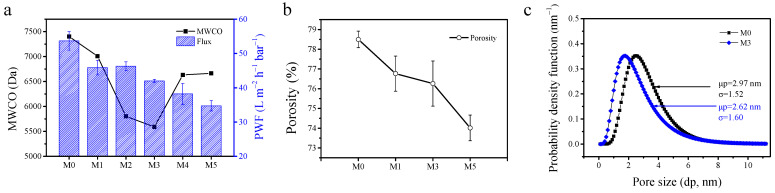
The pure water fluxes and molecular weight cut-off (MWCO) of membranes (**a**); the porosity of M0, M1, M3, and M5 (**b**); the pore size distribution of M0 and M3 (**c**).

A series of PEG (PEG 2000 Da, 4000 Da, 6000 Da, 8000 Da) solutions were used as the model molecules. All membranes showed low MWCO values (<7500 Da), indicating the tight characteristic of these membranes. Moreover, the MWCO of membranes was impacted by the blending of MoS_2_@PDA in casting solutions. When the added concentration of MoS_2_@PDA was 3.0 wt %, the membrane (M3) had the smallest MWCO value as 5589 Da. The major reason for this trend was that with the addition of nanoparticles, the adsorption between the exposed hydroxyl group on the surface of MoS_2_@PDA and the polymer chain increased the viscosity of the casting solution, resulting in delayed phase separation [53,54]. It was beneficial to create a denser structure in MoS_2_@PDA/PES MMM. However, when the concentration of MoS_2_@PDA further increased, the MWCO increased instead, which was related to the agglomeration of nanoparticles on the membrane structure, and the defects deteriorate the PEG rejection. With the increase of MoS_2_@PDA concentration, the pure water flux of the membrane decreased from M0 53.67 ± 2.73 L m^−2^ h^−1^ bar^−1^ to M5 34.75 ± 1.5 L m^−2^ h^−1^ bar^−1^. The reduced water flux was directly related to the decreased porosity with the increasing of MoS_2_@PDA in membranes. With the increase of MoS_2_@PDA loading, the porosity decreased gradually from 78.50 ± 0.42% for the M0 membrane to 74.02 ± 0.65% for the M5 membrane (Figure 9b). Moreover, compared to the M0 membrane, the MoS_2_@PDA blended TUF membrane has smaller pore sizes and MWCO values (Figure 9c). The average pore size of the membrane reduced from 2.97 nm for the M0 membrane to 2.62 nm for the M3 membrane. As a result of its smallest MWCO value, the M3 membrane was selected for further evaluation of dye separation performance.

**Figure 10:**
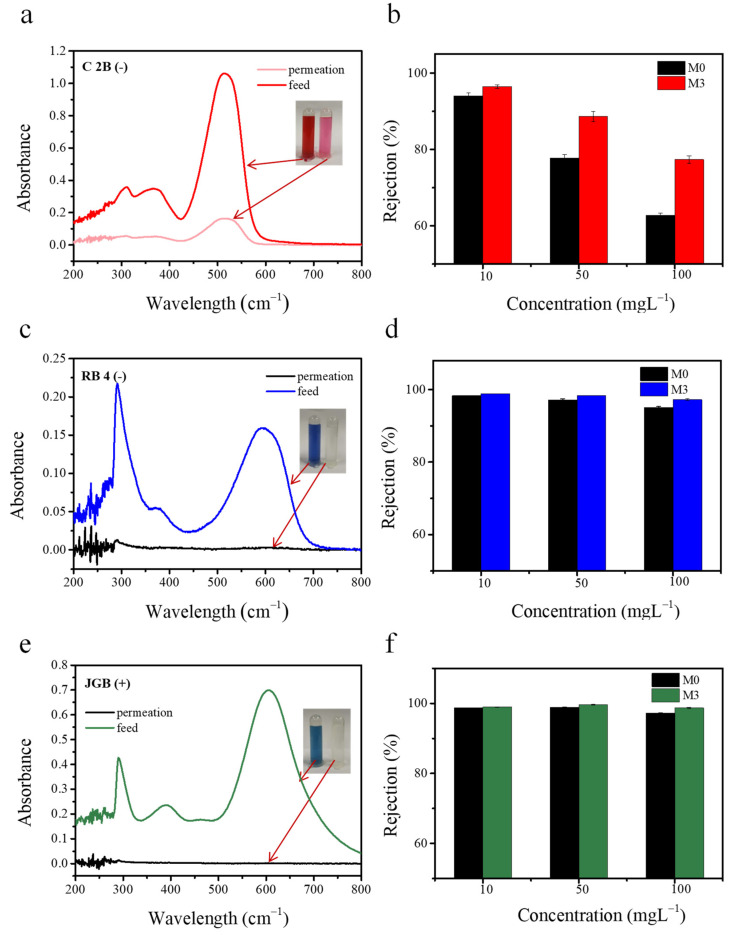
UV-vis absorbtion spectra of the feeds and the permeation of dyes for membrane M3: Chromotrope 2B (C2B) (**a**), Reactive Blue 4 (RB4) (**c**), and Janus Green B (JGB) (**e**). The insets are the corresponding photographs of the dye solutions before and after filtration. Rejection of M0 and M3 membrane for different concentrations of dyes C2B (**b**), RB4 (**d**), and JGB (**f**).

Dye removal experiments were conducted by using three kinds of dyes solution (C2B, RB4, and JGB) with different concentrations (10, 50, and 100 mgL^−1^). Figure 10 shows the retention performance of different dyes. Figure 10a,c,d shows the separation effect of M3 on 50 mgL^−1^ dye, displaying the UV-visible spectra of the dye solution before and after the filtration. The obvious difference of colors of the feed solution and permeate solution indicated the high dye rejection performance of the M3 membranes. Moreover, compared with the pristine M0 membrane, the M3 membrane showed better dye retention effects to those dyes. The RB4 and JGB selectivity performances of the M3 membrane were stable even when the dye concentration increased from 10 to 100 mgL^−1^. However, for the C2B solution, as the dye concentration increased, the membrane retention rate fell from 94.17% to 78.43%. This may be due to the increase of impurities with the increase of dye concentration. The addition of these ions enhanced electrostatic shielding, thus reducing the rejection of the dye [55].

**Figure 11:**
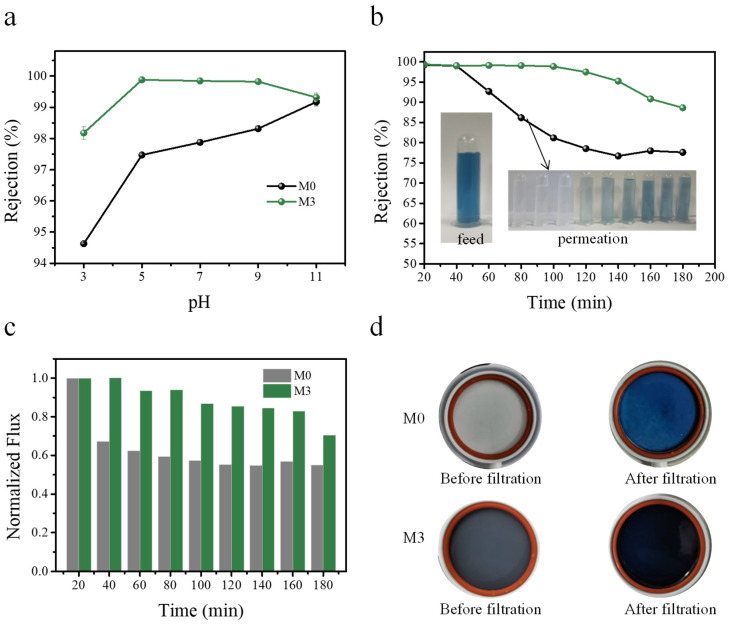
Separation performance of JGB dyes by membrane M0 and M3. Effect of different pH values on the membrane separation performance. (Dye: JGB, pH range: 3–11, and the pH was adjusted with HCl and NaOH) (**a**); effect of filtration time on rejection in pH 11 solution (**b**); effect of filtration time on permeation in pH 11 solution (**c**); M0 and M3 physical images before and after filtering (**d**).

The zeta potential characterization of the membrane is shown in Figure 8a. With the increase of pH, the negative charge on the membrane surface was enhanced, and the zeta potential of the M3 membrane was always lower than that of the M0 membrane. The lower zeta potential for the M3 membrane was more conducive to the adsorption of positively charged dye JGB. Due to the concentration polarization on the membrane surface, the dye retention rate would increase with the increase of pH (Figure 11a) [56]. The membrane’s interception rate of JGB decreased with the extension of filtration time in a longer period of filtration (Figure 11b). As the separation time was extended to 180 min, the JGB rejection of the M3 membrane was 88.61%, while the JGB rejection of the M0 membrane fell to 77.59%, indicating the good stability of the M3 membrane. Figure 11c shows the water flux of the M0 and M3 membranes during the long filtration test. Compared to the M0 membrane, the M3 membrane showed a low water flux reduction rate, which further confirmed the stable dye filtration performance of the M3 membrane. In future work, we will select more types of pollutants with different sizes and involve membrane cleaning cycles to investigate the stability and the service life of the membrane.

**Figure 12:**
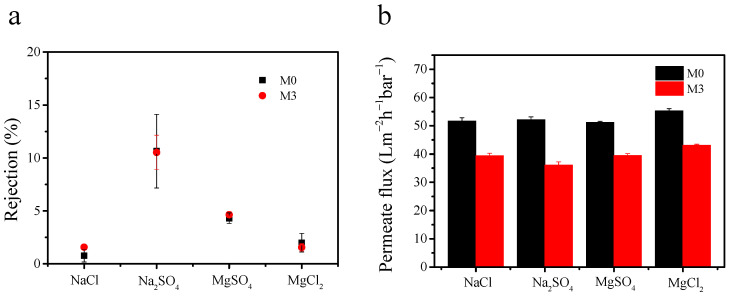
Salts separation performance: rejection measured for different salt solutions (1000 mg/L) (**a**); permeation flux of salt solution (**b**); (pressure of 1 bar was applied).

The high salinity in the textile wastewater affects the retention of dyes by the membrane. Passing the salt through the membrane is a possible strategy to separate dye and salt in one step. This will save costs for subsequent recovery of dye and salt [57]. The filtration performance of the pristine membrane and MoS_2_@PDA-modified membrane was further evaluated with different salts (shown in Figure 12). As expected, the rejection of salts was inefficient, showing 0.76%, 10.64%, 4.31%, and 1.99% for NaCl, Na_2_SO_4_, MgSO_4_, and MgCl_2_, respectively. Accordingly, because M0 and M3 were negatively charged, the low salt rejection could be ascribed to the membrane screening effect. The hydration radius of salt ions was much smaller than the membrane pore size (Na^+^: 0.36 nm, Mg^2+^: 0.43 nm, SO_4_^2−^: 0.38 nm, Cl^−^: 0.33 nm) [58]. The small-sized ions could be allowed to pass through the membrane pores easily [59].

**Figure 13:**
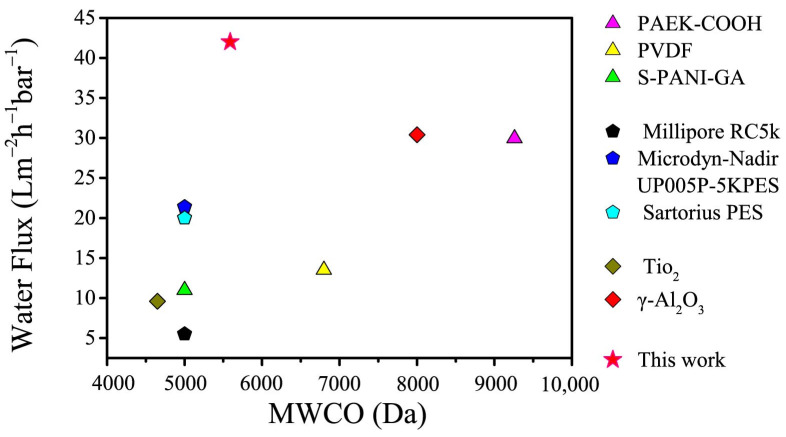
The TUF membranes with similar MWCO are listed. Water flux of non-commercial polymer TUF membranes in the literature [60,61,62], commercial TUF membranes reported in the literature [63,64], ceramic TUF membranes reported in the literature [65,66] and a MoS_2_@PDA/PES mixed matrix TUF membrane in this work are compared.

Figure 13 compared the water flux of the M3 membrane and other TUF membranes with similar MWCO in previous studies. The result indicated that the M3 membrane manufactured in this study had a higher water flux than other TUF membranes with similar MWCO values. All these further revealed that the MoS_2_@PDA-modified TUF has a high feasibility to be applied for dye separation.

## 4. Conclusions

This study developed a one-step phase inversion approach to fabricate MoS_2_@PDA-modified TUF membranes. Improvements of MoS_2_@PDA on the properties and filtration performance were investigated by varied membrane characterization and performance evaluation. The blending of MoS_2_@PDA into PES membranes results in a denser membrane structure and enhanced thermal stability and electronegativity. In addition, TUF membranes with different MWCO were obtained by controlling the loading of MoS_2_@PDA. The membrane with 3.0 wt % MoS_2_@PDA had a lowest MWCO (5589 Da), showed a good pure water permeation flux as 42.0 Lm^−2^h^−1^ bar^−1^, and had a high rejection of dye JBG up to 99.88% at pH 5. The salt ions removal rate of membrane M3 was mostly lower than 10%, indicating that the membrane provided free salt ions permeation. The MoS_2_@PDA/PES membrane prepared in this work has the potential to efficiently separate and recover dye and salt.

## Figures and Tables

**Table 1:** The composition of casting solutions for MoS_2_@PDA/polyethersulfone (PES) tight ultrafiltration (TUF) mixed matrix membranes.

Membrane	PES (wt %)	PVP (wt %)	DMAc (wt %)	MoS_2_@PDA (wt %)
M0	23.0	16.0	61.0	0.0
M1	23.0	16.0	59.0	2.0
M2	23.0	16.0	58.5	2.5
M3	23.0	16.0	58.0	3.0
M4	23.0	16.0	57.0	4.0
M5	23.0	16.0	56.5	4.5
